# Influence of reef habitat on coral microbial associations

**DOI:** 10.1111/1758-2229.70051

**Published:** 2024-11-08

**Authors:** Shelby E. Gantt, Keri M. Kemp, Patrick L. Colin, Kenneth D. Hoadley, Todd C. LaJeunesse, Mark E. Warner, Dustin W. Kemp

**Affiliations:** ^1^ Department of Biology University of Alabama at Birmingham Birmingham Alabama USA; ^2^ Department of Medicine University of Alabama at Birmingham Birmingham Alabama USA; ^3^ Coral Reef Research Foundation Koror Palau Micronesia; ^4^ Department of Biological Sciences University of Alabama Tuscaloosa Alabama USA; ^5^ Dauphin Island Sea Lab Dauphin Island Alabama USA; ^6^ Department of Biology Pennsylvania State University State College Pennsylvania USA; ^7^ School of Marine Science and Policy University of Delaware Lewes Delaware USA

## Abstract

Corals have complex symbiotic associations that can be influenced by the environment. We compare symbiotic dinoflagellate (family: Symbiodiniaceae) associations and the microbiome of five scleractinian coral species from three different reef habitats in Palau, Micronesia. Although pH and temperature corresponded with specific host‐Symbiodiniaceae associations common to the nearshore and offshore habitats, bacterial community dissimilarity analyses indicated minimal influence of these factors on microbial community membership for the corals *Coelastrea aspera*, *Psammocora digitata*, and *Pachyseris rugosa*. However, coral colonies sampled close to human development exhibited greater differences in microbial community diversity compared to the nearshore habitat for the coral species *Coelastrea aspera*, *Montipora foliosa*, and *Pocillopora acuta*, and the offshore habitat for *Coelastrea aspera*, while also showing less consistency in Symbiodiniaceae associations. These findings indicate the influence that habitat location has on the bacterial and Symbiodiniaceae communities comprising the coral holobiont and provide important considerations for the conservation of coral reef communities, especially for island nations with increasing human populations and development.

## Graphical abstract

In Palau, Micronesia, temperature and pH influence coral‐Symbiodiniaceae associations but do not affect coral microbial community membership across nearshore and offshore reef habitats. Coral colonies near human development exhibited higher microbial diversity, greater bacterial community similarity across genera, and less consistent Symbiodiniaceae associations compared to both nearshore and offshore habitats. These findings reveal how habitat and proximity to urban development affect coral symbioses, offering insights for coral reef conservation as human development increases in coastal environments.

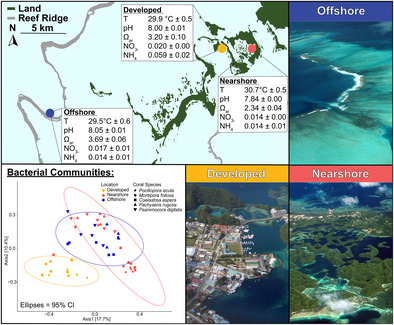

## INTRODUCTION

Corals have a long evolutionary history with their microbial symbionts that has resulted in high levels of microbiome assemblage cophylogeny, or shared phylogenetic history, with the coral host (Pollock et al., 2018; Sunagawa et al., 2010) and has likely contributed to host‐specific microbiomes (Rohwer et al., 2002). Coral‐microbial associations play a crucial role in shaping the physiology and phenotypic characteristics of corals (Bourne et al., 2016). These associations may influence the physiological flexibility of the coral holobiont, consisting of the coral host, photosynthetic endosymbiotic dinoflagellates (from the family Symbiodiniaceae), and various fungi, protists and microbes (Bourne et al., 2009). Consistent and specific core microbial members are typically noted within diverse coral species and habitats (Ainsworth et al., 2015). These essential microorganisms play a crucial role in maintaining the stability and metabolic functionality of the coral holobiont, contributing to the overall health and adaptability of coral communities.

The identification of coral‐associated microbes (i.e., resident bacteria and dinoflagellate symbionts) across different colonies, species, and environments is therefore important in advancing our understanding of the intricate microbial ecology of reef corals (Ainsworth et al., 2015; Pollock et al., 2018). Additionally, understanding these interactions may contribute to coral health and physiological stability. However, environmental influences on microbial community composition can be significant (Hernandez‐Agreda et al., 2018) and understanding these fluctuations may be crucial for elucidating the resiliency of microbial processes important to coral hosts. Gaining such knowledge is essential for predicting how changing environments may affect the overall physiology of coral holobionts.

Abiotic factors influence the membership of coral microbial communities. Many coral microbiota are sensitive to environmental stressors that can generate dysbiosis or altered community states. Warming seawater temperatures are causing more frequent local and world‐wide bleaching events (Hoegh‐Guldberg et al., 2023), which continue to threaten coral reef diversity and ecosystems (Hughes et al., 2018). In populated coastal areas, poor water quality from nutrient pollution and runoff can disturb the diversity and composition of microbial communities on nearshore coral reefs (Littman et al., 2011; Ziegler et al., 2016). Broad changes in coral microbial communities can shift physiological processes for acclimatisation to minimize the effects of environmental stress (Epstein et al., 2019; Kemp et al., 2023; Pantos et al., 2015; Vega Thurber et al., 2009; Ziegler et al., 2017). For these reasons, it is important to consider the environmental factors that may be influencing the structure of coral microbial communities.

The identity of the dominant Symbiodiniaceae species, may also affect the composition of a coral's microbial community (Bourne et al., 2013). Dinoflagellate symbionts have a wide range of physiological abilities (Abrego et al., 2008; Díaz‐Almeyda et al., 2011; Hoadley, Pettay, et al., 2021), which can exert various biotic effects on the host environment. These effects may influence and shape the composition of microbial communities (McIlroy et al., 2020). Constraints to host Symbiodiniaceae associations, such as vertical transmission of Symbiodiniaceae symbionts, may also influence the role microbial communities have for local acclimatisation of the holobiont (Botté et al., 2022). Bacterial symbionts play a crucial role in maintaining the stability of microbial communities and promoting coral health by preventing harmful microbes from colonizing the coral mucus (Krediet et al., 2013). While significant strides have been taken in deciphering microbial interactions and their relative importance to the coral host, there remains uncertainty regarding how life history traits and different associations with Symbiodiniaceae might influence these relationships.

This study compares the diversity of eukaryotic and prokaryotic symbionts across three reef locations in Palau, Micronesia, each with varying seawater properties and proximity to land and urban development. By leveraging natural differences in temperature and urban proximity, this study examines the influence of these reef locations on coral‐Symbiodiniaceae and coral‐bacterial associations. High coral cover and many of the same coral species occur throughout these different reef environments (Barkley et al., 2015; Keister et al., 2023). Five coral species were sampled from an offshore barrier reef and two nearshore lagoon habitats that are on average ~1.0 to 2°C warmer, and ~0.05 to 0.15 pH units more acidic, than the offshore reef due to lower water turnover. The nearshore habitats were further characterized by proximity to anthropogenic development. For each sampled colony, the resident dinoflagellate symbiont and the bacterial consortia were characterized. This diversity was compared across host species and habitats to determine the relative influence of reef location and coral‐Symbiodiniaceae association had on the bacterial assemblages associated with each coral colony. Though different Symbiodiniaceae associations were found across habitats within coral species, multivariable models indicated that dominant Symbiodiniaceae association did not have significant influence on the structure of coral microbial communities. However, microbial community distributions varied considerably across sampled habitats, with the colonies sampled near human developments having the largest shifts in microbial community structure. These findings indicate that, for some coral populations, lower pH and increased seawater temperatures currently have less influence on coral microbial community structure than human proximity. Further, the discoveries from this work emphasizes the importance of conserving coral reef ecosystems, while offering considerations for coastal land development near these habitats.

## EXPERIMENTAL PROCEDURES

### Reef locations and environmental conditions

**FIGURE 1:**
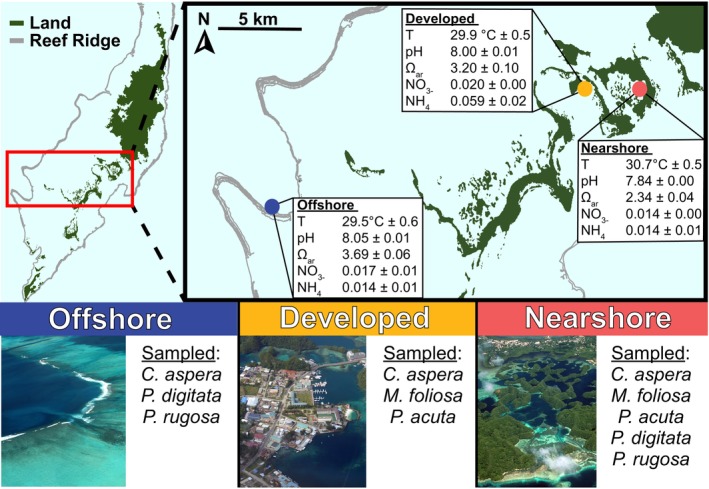
Map of sample habitats with habitat specific information. The reef crest is denoted with grey lines, the water is shown in light blue, and dark green is land. The blue circle indicates the offshore habitat (Rebotel Reef), yellow circle indicates the developed habitat (Malakal Island), and the red circle indicates the nearshore habitat (Ngermid Bay). Habitat parameters are the average ± the standard deviation. Nitrate and ammonium (mg L^−1^) were measured by authors in 2013 for the nearshore and offshore habitats, and in 2022 for the developed habitat. Other habitat parameters were incorporated from Shamberger et al. (2014), Barkley et al. (2015), and Kurihara et al. (2021).

Three different reef habitats were selected based on their proximity to human development and known seawater properties (Barkley et al., 2015; Kurihara et al., 2021; Shamberger et al., 2014). A nearshore reef found in Ngermid Bay (also referred to as Nikko Bay, nearshore, 7° 19.470′ N, 134° 29.634′ E) with no adjacent development (~2 km to development; Figure 1) was selected due to its known warmer temperatures and more acidic seawater chemistry. In contrast, a nearshore reef on Malakal Island (developed, 7° 20.192′ N, 134° 27.546′ E) was selected directly adjacent (<50 m) to development including hotels, restaurants, a boat ramp, and marinas (Figure 1). Finally, Rebotel Reef (offshore, 7° 14.930′ N, 134° 14.149′ E) was chosen as an offshore forereef habitat that is ~28 km from human development (Figure 1) and has cooler seawater temperatures and greater pH than the nearshore reef habitats.

Temperature loggers were deployed in January 2022 and were recovered December 2023 from all reef locations using HOBO Pro v2 underwater temperature loggers (Onset Computer Corporation, Bourne, MA). Loggers were set to sample every 10 min with a resolution of ±0.2°C. Daily means from 1 year of data collection were used for temperature averages (Figure 1). In 2022, water samples from the developed reef habitat were collected for ammonium (NH_4_^+^) and nitrate (NO_3_^−^) analysis, to compare with 2013 offshore and nearshore habitats and previously published water chemistry from surrounding habitats (Barkley et al., 2015; Kurihara et al., 2021; Shamberger et al., 2014). Seawater for all habitats was collected ~1 m above the reef with sterile 50 mL falcon tubes. All seawater nutrient samples were kept frozen until nutrient analysis at the Center for Applied Isotope Studies at the University of Georgia. Aragonite saturation state and pH at sampled habitats were utilised from Shamberger et al. (2014), Barkley et al. (2015), and Kurihara et al. (2021) for habitat characterisation but not subsequent analyses. All data for habitat characterisation are included in Appendices S1 and S2 Palau Temperature and Nutrients Data 2022.xls file. Maps were made using QGIS 3.28.1‐Firenze (https://qgis.org/en/site/) from data collected during a benthic habitat mapping project by the National Oceanic Atmospheric Administration's (NOAA) National Centers for Coastal Ocean Science (NCCOS) (Anderson, 2007; Battista et al., 2007).

Temperature and nutrient statistics were analysed across habitats in R version 4.2.2. Temperature, NH_4_^+^ and NO_3_^−^ were visualised with a histogram and tested for normality with a Shapiro‐Wilks test, while equal variance was tested with a Levene's test. Temperature and NO_3_^−^ met both assumptions and were analysed with a one‐way ANOVA followed by a Tukey honestly significant difference (HSD) test for pairwise comparisons. NH_4_^+^ did not have a normal distribution, even after transformations, so the non‐parametric Kruskal–Wallis test was used to assess significant differences across all habitats, followed by the Dunn's test with Bonferroni correction for pairwise comparisons between habitats.

### Sample collection

Five scleractinian coral species (*Coelastrea aspera*, *Montipora foliosa*, *Pocillopora acuta*, *Pachyseris rugosa* and *Psammocora digitata*), that represent a variety of life history characteristics (Table 1), were sampled on 3–15 June 2022, from the nearshore, developed, and offshore habitats. *C. aspera* was sampled at all habitats (Table 1, Figure 1). *P. acuta* and *M. foliosa* were only sampled at the nearshore and developed habitats, while *P. digitata* and *P. rugosa* were sampled only from the nearshore and offshore habitats (Table 1, Figure 1). Coral fragments were collected (*n* = 4–5 colonies) at 1–5 m depth at the nearshore habitats and 5–10 m depth from the offshore habitat to ensure similar light conditions across habitats as established by Hoadley, Pettay, et al. (2021). Fragments were chiselled from the tops of wild colonies, avoiding epibionts and new growth, placed in individual whirl packs and were immediately flash frozen in liquid nitrogen. Triplicate seawater samples (1 L) were collected from ~1 m above each reef and were filtered through a sterile 47 mm, 0.45 μm cellulose (Millipore) filter before being flash frozen. The seawater was sampled from the developed habitat on one occasion (*n* = 3), while nearshore (*n* = 4) and offshore (*n* = 6) habitats were each sampled on two occasions, around the same time of day during coral collections.

**TABLE 1:** Coral species information and sampling design.

Species	Sex System	Reproduction	Dinoflagellate Transmission	Habitats	Dominant Symbiodiniaceae Symbiont
*P. acuta*	Hermaphroditic	Brood	Vertical	N, D	*D. glynnii*
*M. foliosa*	Hermaphroditic	Spawn	Vertical (eggs)	N, D	*D. glynnii*, *Cladocopium* C15
*C. aspera*	Hermaphroditic	Spawn	Horizontal	N, D, O	*D. trenchii*, *D. trenchii* & *C. madreporum*, *C. madreporum*
*P. digitata*	Dioecious	Spawn	Horizontal	N, O	*D. trenchii*, *C. patulum*
*P. rugosa*	Dioecious	Spawn	Horizontal	N, O	*D. trenchii*, *C. patulum or Cladocopium* C27 and *D. trenchii*

*Note*: Symbiotic dinoflagellate transmission refers to Symbiodiniaceae symbiont acquisition by the host. Habitats are abbreviated as follows: nearshore (N), developed (D) and offshore (O). If more than one dominant Symbiodiniaceae symbiont was found across habitats, then symbionts are listed in the same order as the habitats where sampled. Sex system, reproduction mode, and dinoflagellate transmission were determined from the following publications: Penland et al. (2004), Babcock et al. (1986), Kitchen et al. (2020).

### DNA extraction and sequencing

DNA was extracted from ~2 cm^2^ of coral tissue (avoiding as much skeleton as possible), or half of a 0.45 μm filter for seawater samples, using the ZymoBIOMICS™ DNA Miniprep kit (ZYMO) following manufacturer protocols. Extracts were eluted in 50 μL of DNase/RNase free water and DNA concentrations were quantified using a Qubit™ dsDNA BR Assay kit on a Qubit 4 fluorometer (Invitrogen). Amplicon library preparation and paired‐end sequencing of the 16S rRNA gene V4 variable region was completed using the primers 515F and 806R (Caporaso et al., 2011) with a 50 K read depth per sample on an Illumina Miseq following the manufacturer's guidelines (www.mrdnalab.com, Shallowater, TX).

Palau corals maintain specific and stable Symbiodiniaceae associations through time with consistent within colony homogeneity (Lewis et al., 2024). Therefore, Symbiodiniaceae community composition was determined with DNA extracts using *Cladocopium* and *Durusdinium* genera‐specific actin gene primer pairs (McGinley, 2012) with quantitative PCR (qPCR) on a QuantStudio 3 (Applied Biosystems) as described in Gantt et al. (2023). Briefly, DNA was standardized to 10 ng/μL before qPCR amplification, absolute quantification (Mieog et al., 2009) was used to generate estimates of community proportions, and genus‐specific standard curves were used to estimate the number of Symbiodiniaceae cells per sample well. The abundance of the Symbiodiniaceae symbiont by genera (*Cladocopium* and *Durusdinium*) relative to total Symbiodiniaceae was calculated as in Gantt et al. (2023). Sub‐genus identification of dominant Symbiodiniaceae community members were completed using internal transcribed spacer 2 (ITS2) of the rRNA gene region with ITS2intfor and ITS2rev primers (LaJeunesse, 2002). Final ITS2 amplicons were cleaned with the Exo‐CIP^TM^ PCR Cleanup kit and sanger sequenced at the Heflin Center for Genomic Sciences at the University of Alabama at Birmingham. Final sequences were pairwise aligned using Geneious Prime® version 2022.1.1. Sequences for symbiont ITS2 identification are archived at GenBank accession numbers PP391597 – PP391614 and PP769353 – PP769369.

### 16S rRNA amplicon sequence quality control and initial processing

Initial 16S rRNA amplicon processing was completed using the MR DNA ribosomal and functional gene analysis pipeline (www.mrdnalab.com, MR DNA, Shallowater, TX). During this processing, Illumina adapters were removed from raw sequences and sequences <150 bp in length or with ambiguous base calls were removed. Further processing of amplicon libraries was completed in R (version 4.2.2) as described in Kemp et al. (2021). Briefly, the DADA2 package (version 1.26.0) (Callahan, McMurdie, et al., 2016) was used to determine exact amplicon sequence variants (ASVs) from raw reads, as described by Callahan, Sankaran, et al. (2016). DADA2 was also used for quality filtering (removing ASVs with <3 counts per sample and that were present in <5 samples), followed by dereplication, error estimation, ASV inference, and merging of paired reads. Final assignment of taxonomy to ASVs was completed with the SILVA small subunit ribosomal RNA database (v138). DADA2 was also used with the ‘addSpecies’ command for ASV species level assignment with 100% identity matching. The ASV and taxonomy tables and the metadata were imported into phyloseq version 1.42.0 (McMurdie & Holmes, 2013) for visualisations and statistical analyses. Sequences not identified as bacteria or archaea and sequences that identified as chloroplasts or mitochondria were removed from the dataset. One nearshore *C. aspera* and one nearshore *P. acuta* were removed because their communities did not rarefy. Additionally, one developed habitat *M. foliosa* was identified as an outlier and was removed after identification from Shannon‐Weaver and Simpson alpha diversity measures via boxplots and the Grubb's test (*p* < 0.05) from the outliers package (Komsta & Komsta, 2007). Microbial community sequences are archived at SRA accession number PRJNA1080224.

### Microbial alpha diversity

Samples were rarefied to the lowest read count (3548 reads) before analyses. Simpson, Shannon‐Weaver, and Chao1 diversity indices were calculated with the *estimate_richness* function from the phyloseq package (McMurdie & Holmes, 2013). Normality of alpha diversity data was assessed using histograms and Shapiro–Wilks tests and equal variance with a Levene's test. All Shannon values were antilog transformed to achieve normality. Significant differences in alpha diversity across reef habitats for seawater and *C. aspera* samples was assessed with one‐way ANOVAs followed by post‐hoc pairwise comparisons via Tukey HSD. Kruskal–Wallis tests followed by post‐hoc Dunn's tests with Bonferroni correction for multiple comparisons were used for Simpson indices, which were not normal after transformation but had similar distributions. For all other coral species, which were sampled at two reef habitats, significance was assessed with Student's *t*‐tests or non‐parametric Mann–Whitney *U*‐tests.

### Microbial community composition (beta diversity)

Rarified ASV count tables were centered log‐ratio transformed and used to generate Bray–Curtis dissimilarity matrices for Principal Coordinate Analysis (PCoA). A global permutational analysis of variance (PERMANOVA) was performed with adonis2 (999 permutations, Euclidean distance) from the vegan package version 2.6‐4 (Oksanen et al., 2013) with the formula beta diversity ~ coral species * habitat. Follow‐up pairwise permutations were conducted with the function *pairwise.adonis2* to assess the association between habitat and beta diversity of individual coral species or seawater (Martinez Arbizu, 2020), with a Bonferroni correction to control for multiple pairwise comparisons.

### Differential abundance testing of microbial taxa

Differential abundance was determined across coral species via the microbiome multivariable associations with linear models (MaAsLin2) package (Mallick et al., 2021), a robust and consistent method for analysing differential abundances (Nearing et al., 2022), in R (version 4.2.2). MaAsLin2 tests were run with rarified counts after zero ASVs were removed for each level of analysis (Phylum, Family and Genus). Analysis was conducted using the NEGBIN method and counts were Trimmed Mean of M component (TMM) normalised. No transformations were performed with the analysis, a minimum prevalence of 0.10 was used along with a minimum abundance of 20, the Benjamini‐Hochberg (BH) method for bias correction of false discovery rate was applied with a maximum significance *q*‐value of 0.05. To identify ‘habitat‐associated’ coral taxa that changed between reef habitats in a similar manner across all coral species examined, MaAsLin2 was used while adjusting for coral species as follows: offshore versus nearshore habitats, adjusting for coral species (*C. aspera*, *P. digitata*, *P. rugosa*), and developed versus nearshore habitats, adjusting for coral species (*C. aspera*, *M. foliosa*, *P. acuta*), with the nearshore habitat as the reference for each comparison. To determine habitat effects within a given sample type, separate follow‐up MaAsLin2 models were run for seawater samples and each coral species with reef habitat as the fixed effect.

## RESULTS

### Reef habitat temperature, nitrate, and ammonium comparison

Temperature differences across habitats were significant, with each habitat significantly distinct from the others (One‐way ANOVA and Tukey HSD, all *p* < 0.001). NH_4_^+^ significantly differed across habitats (Kruskal–Wallis, *p* = 0.013), and was four times greater at the developed habitat (0.059 ± 0.023 SD mg L^−1^, Figure 1) than the nearshore habitat (0.014 ± 0.005 SD; Dunn's test, *p* = 0.036, Figure 1) and offshore habitat (0.014 ± 0.011 SD mg L^−1^; Dunn's test, *p* = 0.019, Figure 1). NH_4_^+^ (Dunn's test, *p* = 1.00) was statistically similar between the offshore and nearshore habitats. NO_3_^−^ did not significantly differ across habitats (One‐way ANOVA, *p* = 0.4112), even though the highest average concentrations were at the developed habitat (0.02 ± 0.001 SD mg L^−1^; Figure 1) rather than the nearshore (0.014 ± 0.005 SD mg L^−1^, Figure 1) or offshore habitats (0.017 ± 0.009 SD mg L^−1^; Student's t‐test, *p* = 0.012, Figure 1).

### Symbiodiniaceae identification and community composition

**FIGURE 2:**
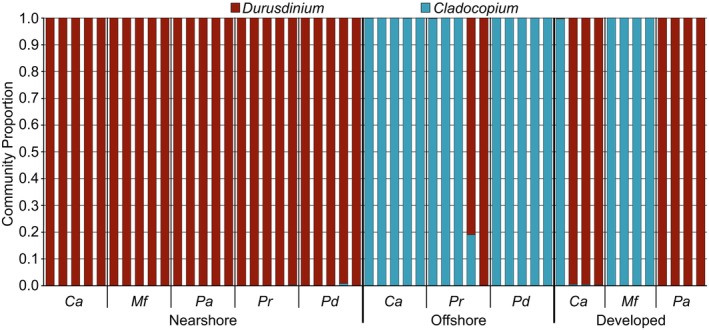
Real‐time PCR estimates of genus‐level Symbiodiniaceae community relative proportions by coral individual. Data are organized by sampled habitat and then coral species. Coral species are as follows: *C. aspera* (Ca), *M. foliosa* (Mf), *P. acuta* (Pa), *P. rugosa* (Pr), *P. digitata* (Pd). Bars represent distinct coral genets (*n* = 4–5) from each habitat.

Almost all offshore coral species had *Cladocopium* spp. symbionts, except for two *P. rugosa* colonies that had mixed communities of *D. trenchii* and *Cladocopium patulum* (Butler et al. (2023), previously known as type C3u, Figure 2, Table 1). *Durusdinium* spp. were the dominant symbionts associated with all individuals across all coral species at the nearshore habitat. Specifically, *D. glynnii* associated with *P. acuta* and *M. foliosa*, while *D. trenchii* was found in *C. aspera*, *P. digitata*, and *P. rugosa*. At the developed habitat, *M. foliosa* associated with *Cladocopium* C15, *C. aspera* associated with either *Cladocopium madreporum* or *D. trenchii*, and *P. acuta* associated with *D. glynnii*.

### Microbial alpha diversity

There were no consistent associations among habitats and alpha diversity across coral species. The Simpson diversity index differed significantly across *C. aspera* microbial communities from all habitats (Kruskal–Wallis test, *p* = 0.0369, Table 2), and was driven by significant differences between the offshore and developed habitats (Dunn's test, *p* = 0.0346), which had the lowest and highest indices, respectively (Table 2). Microbial diversity was similar for *C. aspera* when comparing the nearshore and offshore and the nearshore and developed habitats (Dunn's test, *p* = 0.2536 and 1.000, respectively). Microbial diversity was greater for nearshore *P. acuta* than at the developed habitat (Mann–Whitney test, *p* = 0.0286, Table 2). Only *P. rugosa* had significant differences in Chao1, which was greater in nearshore than offshore habitats (Student's *t*‐test, *p* = 0.0246, Table 2). Microbial diversity of seawater differed significantly by habitat for Shannon‐Weaver diversity (One‐way ANOVA, *p* = 0.0021, Table 2) and Chao1 (One‐way ANOVA, *p* = 0.0021, Table 2). Significant differences in Chao1 for seawater were found between all habitat pairwise comparisons (Tukey HSD, Offshore:nearshore *p* = 0.0054; nearshore:developed *p* = 0.0002; and offshore:developed *p* = 0.0169), while the offshore habitat drove significant differences in Shannon‐Weaver diversity for the nearshore (Tukey HSD, *p* = 0.0478) and developed (Tukey HSD, *p* = 0.0018) habitat seawater comparisons.

**TABLE 2:** Mean ± standard deviation of microbial alpha diversity across habitats.

Alpha diversity	Habitat	Coral species					
		*P. digitata*	*P. rugosa*	*C. aspera*	*M. foliosa*	*P. acuta*	Seawater
Chao1	Offshore	587 ± 161	456 ± 63.9	384 ± 224	–	–	427 ± 30.3^A^
	Nearshore	494 ± 76.6	586 ± 83.7	526 ± 102	354 ± 38.1	314 ± 22.9	280 ± 88.6^B^
	Developed	–	–	600 ± 282	467 ± 184	283 ± 234	560 ± 49.4^C^
	*p*‐Value	0.2742^1^	**0.0246** ^1^	0.3518^2^	0.2113^1^	0.8004^1^	**0.0021** ^2^
Shannon‐Weaver	Offshore	4.0 ± 1.3	3.9 ± 0.9	2.6 ± 2.3	–	–	2.8 ± 0.2^A^
	Nearshore	3.7 ± 1.1	4.2 ± 0.5	4.2 ± 0.7	4.2 ± 0.1	4.5 ± 0.2	3.2 ± 0.0^B^
	Developed	–	–	5.0 ± 0.1	3.8 ± 0.7	3.9 ± 0.6	3.5 ± 0.3 ^B^
	P‐value	0.4073^1^	0.4314^1^	0.0561^2^	0.2017^1^	0.1474^1^	**0.0021** ^2^
Simpson	Offshore	0.86 ± 0.2	0.86 ± 0.1	0.55 ± 0.5 ^A^	–	–	0.77 ± 0.0
	Nearshore	0.84 ± 0.2	0.92 ± 0.1	0.91 ± 0.1^AB^	0.95 ± 0.0	0.97 ± 0.0	0.82 ± 0.0
	Developed	–	–	0.98 ± 0.0 ^B^	0.86 ± 0.1	0.94 ± 0.0	0.88 ± 0.1
	*p*‐Value	0.4206^3^	0.5476^3^	**0.0369** ^4^	0.1429^3^	**0.0286** ^4^	0.0587^3^

*Note*: Bold *p*‐values indicate significance. ‘–’ indicates no samples collected at this habitat. Superscript letters indicate significant groupings based on Tukey HSD pairwise comparisons for *C. aspera* and seawater.

^1^ Student's *t*‐test.

^2^ One‐way analysis of variance (ANOVA).

^3^ Mann–Whitney *U*‐test.

^4^ Kruskal–Wallis test.

### Microbial community composition (beta diversity)

**FIGURE 3:**
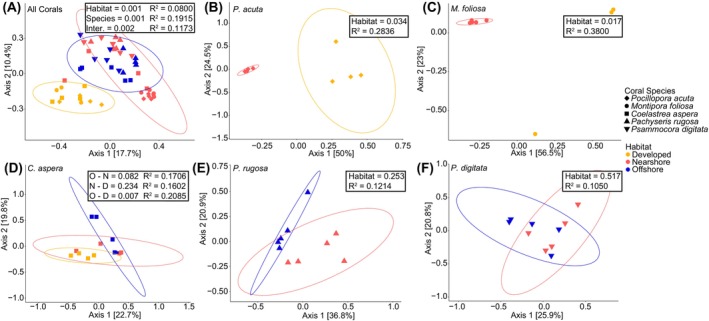
PCoA of Bray‐Curtis dissimilarities of coral microbial communities by coral species and across habitats. The microbial community comparisons are as follows: (A) all corals, (B) *P. acuta* only, (C) *M. foliosa* only, (D) *C. aspera* only, (E) *P. rugosa* only, and (F) *P. digitata* only. Coral species are indicated as follows: *P. acuta* (diamond), *M. foliosa* (circle), *C. aspera* (square), *P. rugosa* (upward triangle), and *P. digitata* (downward triangle). The habitats are indicated as follows: developed (D, yellow), the nearshore (N, red), and the offshore (O, blue). Values indicate significance of groupings by habitat with permutational analysis of variance (PERMANOVA) for panel A and pairwise PERMANOVAs for panels (B)–(F). *M. foliosa* did not have enough individuals from the developed habitat for ellipse creation.

Microbial communities significantly differed by coral species (PERMANOVA, *p* = 0.001, *R* ^2^ = 0.1915, Figures 3A and S1), habitat (PERMANOVA, *p* = 0.001, *R* ^2^ = 0.0800, Figure 3A, *R* ^2^ = 0, Figure S1), and the interaction of these factors (PERMANOVA, *p* = 0.001, *R* ^2^ = 0.1173, Figure 3A). Microbial community composition significantly differed between the developed and nearshore habitats for *P. acuta* (pairwise PERMANOVA, *p* = 0.026, *R* ^2^ = 0.0.2836, Figure 3B) and *M. foliosa* (pairwise PERMANOVA, *p* = 0.021, *R* ^2^ = 0.3800, Figure 3C). No differences in microbial community composition were detected between the offshore and nearshore habitats for *C. aspera* (*p* = 0.082, *R* ^2^ = 0.1706), *P. rugosa* (*p* = 0.263, *R* ^2^ = 0.1214), and *P. digitata* (*p* = 0.508, *R* ^2^ = 0.1050) by pairwise PERMANOVA (Figure 3D–F). *Coelastrea aspera* did not have significant differences in microbial community structure between developed and nearshore habitats (pairwise PERMANOVA, *p* = 0.234, *R* ^2^ = 0.1602, Figure 3D), but did have significant differences between developed and offshore (pairwise PERMANOVA, *p* = 0.007, *R* ^2^ = 0.2085, Figure 3D) habitat comparisons. Seawater microbial community compositions (Figure S2) differed between the developed habitat and the nearshore (pairwise PERMANOVA, *p* = 0.028, *R* ^2^ = 0.5518) and offshore habitats (pairwise PERMANOVA, *p* = 0.032, *R* ^2^ = 0.3080), with no differences detected between the offshore and nearshore habitats (pairwise PERMANOVA, *p* = 0.072, *R* ^2^ = 0.2336).

### Differential abundance of microbial taxa across habitats

**FIGURE 4:**
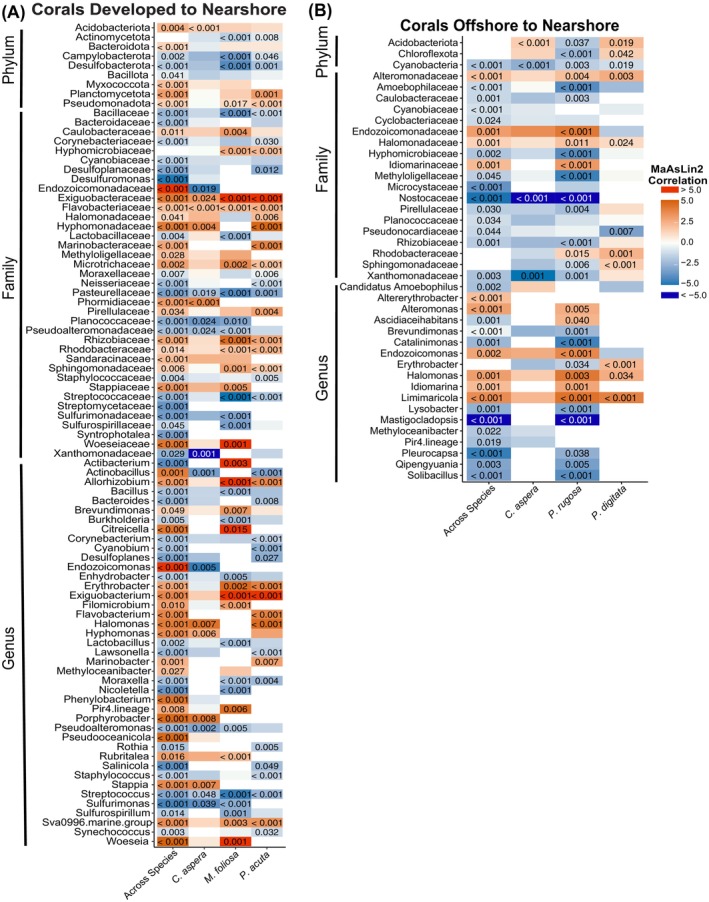
Heat maps of microbial taxa from MaAsLin2 results that significantly differed by coral species with the nearshore habitat as the reference. Taxa are broken up by taxonomy level analysis with results shown for all corals and within each coral species across that habitat comparison. (A) Microbial taxa that were differentially abundant in developed habitat compared to nearshore habitat corals, (B) microbial taxa that were differentially abundant in offshore habitat compared to nearshore habitat corals. Red indicates taxa that are more abundant than at the nearshore habitat (reference), white indicates no difference in abundance, and blue indicates taxa that are less abundant than at the nearshore habitat. FDR controlled *q*‐values greater than 0.05 are not included in the figure for clarity, while *q*‐values lower than 0.001 are indicated with ‘<0.001’.

*Developed Habitat to Nearshore Habitat Coral Comparison*. The phyla Acidobacteriota, Pseudomonadota (previously known as Proteobacteria) and Planctomycetota were significantly lower across all coral species (*C. aspera*, *M. foliosa* and *P. acuta*) at the nearshore than developed habitat (MaAsLin2, *q* = 0.044, <0.001 and <0.001, respectively, Figure 4A), while the phyla Campylobacterota, Desulfobacterota, and Bacillota were significantly higher across all nearshore coral species (MaAsLin2, *q* = 0.002, <0.001 and 0.041, respectively, Figure 4A), as indicated by multivariable MaAsLin2 models, adjusted for reef habitat and coral species. Thirty‐six families also significantly differed across all coral species, with 17 families having significantly higher prevalence nearshore than at the developed habitat, while 41 genera also significantly differed across all coral species between nearshore and developed habitats (Figure 4A). The genus *Endozoicomonas* was significantly lower in *C. aspera* colonies in the developed habitat than in the nearshore habitat (MaAsLin2, *q* = 0.005, Figure S4).

*Offshore Habitat to Nearshore Habitat Coral Comparison*. Across all coral species, the phylum Cyanobacteria was significantly less abundant offshore than nearshore (MaAsLin2, *q* < 0.001 Figure 4B). Seventeen families varied across the offshore to nearshore habitat comparison, with 4 families more abundant offshore (MaAsLin2, *q* < 0.02, Figure 4B) and 13 families lower in abundance offshore than at the nearshore habitat (MaAsLin2, *q* < 0.045, Figure 4B). The family Endozoicomonadaceae and genus *Endozoicomonas* had significantly higher abundances for offshore *P. rugosa* (and trends of higher abundances for *C. aspera*) than in the nearshore habitat (MaAsLin2, *q* < 0.001, Figure 4B). The genera *Altererythrobacter*, *Alteromonas*, *Endozoicomonas*, *Halomonas*, *Idiomarina*, and *Limimaricola* were more abundant offshore than nearshore across all coral species (MaAsLin2, *q* ≤ 0.002, Figure 4B). The genera *Candidatus Amoebophilus*, *Ascidiaceihabitans*, *Brevundimonas*, *Catalinimonas*, *Lysobacter*, *Mastigocladopsis*, *Methyloceanibacter*, *Pir4 lineage*, *Pleurocapsa*, *Qipengyuania*, and *Solibacillus* were more abundant nearshore than at the offshore habitat (MaAsLin2, *q* < 0.02, Figure 4B).

Within coral species across habitat comparisons, the phyla Acidobacteriota and Chloroflexota had species‐specific differences with *C. aspera* and *P. digitata* having greater abundances of this phyla offshore than nearshore (MaAsLin2, *q* < 0.04, Figure 4B), and lower abundances within *P. rugosa* communities offshore (MaAsLin2, *q* < 0.04, Figure 4B). At the family level, increased abundances of Rhodobacteraceae and Sphingomonadaceae were found within *P. digitata* communities at the offshore habitat, while *P. rugosa* had greater abundance of Rhodobacteraceae and lower abundances of Sphingomonadaceae offshore (MaAsLin2, *q* < 0.02, Figure 4B) than nearshore. The genus *Erythrobacter* was more abundant for *P. rugosa* at the nearshore habitat (MaAsLin2, *q* < 0.04, Figure 4B) with similar trends observed for *C. aspera*.

*C. aspera comparison across habitats*. The only coral that was sampled across all three habitats was *C. aspera* (Figures 1 and S4), and microbial community composition significantly differed between the offshore and developed habitats (pairwise PERMANOVA, *p* = 0.044, Table 2). In particular, the phylum Acidobacteria was significantly higher near human development than nearshore (Figure S4), while Pseudomonadota was significantly lower in *C. aspera* microbial communities offshore than at the nearshore habitat (Figure S4). Ten families varied in abundance across habitats for *C. aspera*, with 6 families significantly lower at the developed habitat than nearshore (Figure S4) and 4 families with higher abundances near the developed habitat than nearshore (Figure S4). The genus *Endozoicomonas* was significantly more abundant within nearshore communities than at the developed habitat (Figure S4). Most taxa identified as significantly different across habitats at the genera level were due to large differences in the microbial communities between *C. aspera* colonies from the nearshore and developed habitats (Figure S4).

*Seawater comparisons*. Few taxa varied significantly by habitat within the seawater microbial communities. Two phyla significantly differed across habitats with Bacteriodota having higher abundance and Actinomycetota having lower abundance at the developed habitat than offshore or nearshore habitats (Figure S3). A total of 11 families significantly varied across habitats, with 7 families having higher abundance nearshore and offshore than at the developed habitat (Figure S3). The genera *Candidatus Actinomarina* and *Synechoccus* were significantly higher nearshore than at the developed habitat (MaAsLin2, *q* < 0.001, Figure S3). *HIMB11*, *Marinoscillum*, *NS42b marine group*, and *NS4 marine group* had significantly higher abundance at the developed habitat than nearshore (MaAsLin2, *q* < 0.05, Figure S3). The genera *Clade Ib*, *Cyanobium*, *OM60*, and the *Sva0996 marine group* had lower abundance nearshore than within the developed habitat (MaAsLin2, *q* < 0.05, Figure S3).

## DISCUSSION

Environmental factors and biotic interactions play a crucial role in shaping the composition of dinoflagellate symbionts and bacterial communities within coral colonies. Sampling colonies from multiple coral species across distinct habitats highlights how factors like temperature, acidity, and the influence that human development has on seawater can shape animal‐microbe associations. We confirm previous findings that coral colonies in nearshore reefs of Ngermid Bay, which acquire their Symbiodiniaceae through horizontal transmission, predominantly associate with *Durusdinium trenchii*, while conspecific colonies found offshore at Rebotel Reef primarily associate with *Cladocopium* spp. (Hoadley et al., 2019; Kemp et al., 2023; Lewis et al., 2024). While seawater temperatures and pH seemed to influence the dominance of Symbiodiniaceae symbionts between Ngermid Bay and Rebotel reef colonies, they did not influence the bacterial communities. Interestingly, the direct proximity to development and enriched ammonium of the developed reef habitat (based on specific bacteria taxa) correlated more with changes to the composition of coral associated bacterial communities (Figures 2, 3, 4). At the developed habitat bacterial community membership was greatly influenced, with more similarity in bacterial communities observed across coral genera within this habitat than to conspecifics from other habitats. In contrast, the bacterial communities of colonies from Ngermid Bay and Rebotel Reef remained consistent and relatively host‐specific across nearshore and offshore habitats, likely due to prolonged exposure to these conditions and minimal influence from human development. Our findings indicate that environmental conditions at each habitat differentially influenced the Symbiodiniaceae associations and bacterial communities of these corals.

Coral‐Symbiodiniaceae combinations can differ across small spatial scales affected primarily by the prevailing light and temperature conditions of a given habitat (Bongaerts et al., 2010; Kriefall et al., 2022; LaJeunesse et al., 2004). Coral colonies from the nearshore Ngermid Bay and offshore habitats of Palau maintained different Symbiodiniaceae associations, but there were no significant differences in microbial community structure or diversity associated with these different dominant symbionts (Figure S5). *Psammocora digitata* and *Pachyseris rugosa* both predominantly associated with *Cladocopium patulum* (previously type C3u, Butler et al., 2023) at the offshore habitat, with one *P. rugosa* associated with *Cladocopium* C27, and *Durusdinium trenchii* at the nearshore Ngermid Bay habitat, while *Coelastrea aspera* associated with *Cladocopium madreporum* (Butler et al. (2023), previously known as type C40) at the offshore habitat and *D. trenchii* at the two nearshore habitats (Ngermid Bay and Malakal Island). *Pocillopora acuta* and *Montipora foliosa* both associated with *Durusdinium glynnii* at the nearshore Ngermid Bay habitat. Dominance of *Durusdinium* spp. in Ngermid Bay corals may be due to increased temperatures and more acidic conditions, as *D. glynnii* and *D. trenchii* tend to be more tolerant to physiological stress (Hoadley et al., 2019; Kemp et al., 2023; Turnham et al., 2023). Meanwhile, *M. foliosa* colonies near urban development associated with an undescribed species from the C15‐radiation of *Cladocopium*, a lineage likely specific to *Montipora* hosts (Lewis et al., 2024). Most of the corals sampled had homogenous Symbiodiniaceae associations, which is observed for some Indo‐Pacific coral species that maintain relatively stable Symbiodiniaceae associations over several years (Lewis et al., 2024). These patterns in host‐symbiont combinations are consistent with established knowledge that habitat also strongly influences the dominance of particular Symbiodiniaceae taxa from among a small subset of Symbiodiniaceae species that are compatible with a specific coral host (LaJeunesse, 2020). Thus, variation in Symbiodiniaceae associations observed across the sampled habitats of the current study are an ecological response to prevailing environmental factors (temperature, light, etc.), involving symbionts adapted to particular host taxa and the physical conditions of the habitats where these colonies were obtained.

Coral microbial community composition and structure often shift when exposed to elevated temperatures or changes in pH (Maher et al., 2020; Meron et al., 2012; Morrow et al., 2014). Such fluctuations in temperature or pH possibly modify the chemical composition of the coral's mucus (Lee et al., 2016), thereby altering the microbial community and likely holobiont functioning as well. Similar to how Symbiodiniaceae associations differ between the offshore habitat and the warmer, more acidic nearshore habitat, we expected microbial associations to differ as well; however, no consistent differences in alpha or beta microbiome diversity were found within or across coral species for these two habitats. Nearshore and offshore coral colonies in Palau are therefore likely able to maintain homeostasis and the stability of their microbial associations through acclimatisation or adaptation. For example, these same coral populations possess similar skeletal density, linear extension, and calcification rates (Barkley et al., 2015), and maintain similar energy reserves (Keister et al., 2023) across the disparate environmental characteristics of the nearshore and offshore habitats. Colony growth and energy reserves at these habitats may, therefore, mitigate the influence of environmental characteristics through holobiont plasticity and acclimatisation. However, coral populations adjacent to human development were the only colonies found to have unique microbiomes and differed considerably from conspecifics at the nearshore (Ngermid Bay) and (for *C. aspera*) offshore habitats, including microbial taxa such as *Allorhizobium, Staphylococcus*, and *Halomonas*. Notably, the environmental conditions associated with the habitat near human development had higher ammonium concentrations than the other locations which corresponds with human‐induced nutrient enhancement. Although these findings are from a single sampling timepoint, the concentrations are four times greater than previously sampled conditions found in Ngermid Bay and Rebotel Reef and what has been reported in the literature for surrounding habitats (Barkley et al., 2015; Kurihara et al., 2021; Shamberger et al., 2014). Our findings suggest that colonies may have greater coral‐bacterial microbiome stability and structure to moderately increased temperatures and acidity at the nearshore habitat, but these properties are disrupted when colonies are directly adjacent to urban development. Therefore, local anthropogenic stressors may pose an immediate threat to coral reefs that are also facing anthropogenic climate change in the coming decades.

### Coral microbial taxa shifts across offshore and nearshore habitats

There were a few taxa‐specific shifts in abundances within coral microbial communities across the nearshore and offshore habitats. The phyla Cyanobacteria was lower in abundance within offshore colonies than nearshore Ngermid Bay colonies. The differences in Cyanobacteria abundance (across all corals) among reef populations could be due to higher prevalence of these taxa at the nearshore habitat (Figure S3), or may indicate different feeding preferences for picoplankton, as observed in thermally stressed corals (Hoadley, Hamilton, et al., 2021; Meunier et al., 2019; Tong et al., 2021). The genus *Altererythrobacter* was enriched in nearshore *P. rugosa. Altererythrobacter* are known to proliferate and can inhibit other bacteria at higher temperatures (Guo et al., 2022; Li et al., 2021). *Brevundimonas* (class Alphaproteobacteria), was also in higher abundance within nearshore than offshore colonies, with significant enrichment in *P. rugosa* (nearshore), *M. foliosa* (nearshore), and *C. aspera* (nearshore and developed) microbial communities. Previously, *Brevundimonas* has been found associated with acroporids (Littman et al., 2009) and is linked to environmental nutrient cycling, so this species may provide avenues for nutrient cycling within coral microbial communities.

A common coral symbiont that can co‐evolve with the host (Pollock et al., 2018), *Endozoicomonas*, often maintains species‐specific associations with corals (Hochart et al., 2023). Recent work suggests this genus may synthesize vitamins and amino acids for the coral holobiont that are not made by the host coral (Hochart et al., 2023; Maire et al., 2023). Additionally, higher *Endozoicomonas* abundance is positively correlated with coral growth, but also positively correlated with increased disease susceptibility (Epstein et al., 2023) and reduced holobiont phenotypic plasticity (Pogoreutz et al., 2018; Pogoreutz & Ziegler, 2024). *Endozoicomonas* had significantly higher abundances in offshore than nearshore corals. Since coral microbial community diversity, richness, and structure shifted only slightly across the disparate environments of the nearshore and offshore habitats, these holobionts likely can mitigate or limit the influence of increased acidity and seawater temperatures on their microbial symbionts through phenotypic plasticity and local acclimatisation.

### Coral microbial taxa shifts across developed and nearshore habitats

Some members within the phylum Planctomycetota engage in nitrogen cycling through anaerobic ammonia oxidation or annamox (Kartal et al., 2012; Strous et al., 1999) and are critical for carbon cycling (Glöckner et al., 2003). The genus *Allorhizobium* (family Rhizobiaceae) also has known nitrogen fixers and is found associated with tropical legumes (de Lajudie et al., 1998; Kuykendall & Dazzo, 2015). Both of these taxa were enriched across the coral microbial communities near human development unlike those sampled at the nearshore Ngermid Bay habitat. While not well‐documented within the coral microbiome, the genus *Allorhizobium* was exclusively enriched within coral microbial communities rather than in the seawater from the developed habitat. High prevalence of *Marinobacter* (family Alteromonadaceae) was found across coral species within the developed habitat. This genera can associate with dinoflagellate cultures (Maire et al., 2021) and deep‐water corals, where it has the ability to tolerate and degrade hydrocarbons (Thompson & Gutierrez, 2021) commonly found in water runoff and this occurrence may be the result of the close proximity of a boat ramp and marina fuel docks only a few meters from the sampled colonies at the developed habitat (MacKenzie, 2008; Richmond et al., 2019).

Across all coral species at the developed habitat there were shifts in abundances of known disease mitigating microbial symbionts. For example, *Exiguobacterium* (family Bacillaceae), a highly versatile genera with members that thrive in a wide range of pH, salinity, and temperatures (Zhang et al., 2021), was enriched within the developed coral microbial communities (Krediet et al., 2013). The significantly increased abundance of the known hydrocarbon degrader (Silva et al., 2021) and disease mitigating (Pereira et al., 2017) genus *Erythrobacter*, along with the lower abundances of the phylum Actinomycetota, known to form defensive symbioses with hosts (van Bergeijk et al., 2020), were noted within *M. foliosa* and *P. acuta* microbial communities near human development. There was also increased abundances of Rhodobacteraceae near development for these coral species. Increased prevalence of taxa from the family Rhodobacteraceae has been found associated with diseased coral tissues (Roder et al., 2014; Séré et al., 2014) and is connected to the effects of sedimentation and sewage waste on coral microbial communities (Ziegler et al., 2016). The genus *Pseudoalteromonas* was lower in all colonies and specifically within *C. aspera* and *M. foliosa* at the developed habitat compared to the nearshore habitat. The genera *Pseudoalteromonas* has been linked to antimicrobial activity within the coral holobiont (Shnit‐Orland et al. 2012), is a suspected cue for coral metamorphosis (Alker et al., 2020), and has been hypothesized to remove cadmium from host tissues (Sabdono, 2011). Within developed habitat seawater there was increased presence of *Vibrio* spp., known to cause coral diseases (Munn, 2015; Rosenberg & Falkovitz, 2004), and higher prevalence of the genus *Stappia*, linked to black band disease (Henao et al., 2017). While these colonies showed no signs of disease at the time of sampling, there are likely limits to how much shifts in microbial communities can buffer against anthropogenic and climactic stressors without compromising holobiont health, especially given the differences in seawater conditions at the developed reef.

### Influence of life history characteristics

Life history characteristics are another factor that affects host‐symbiont specificity and likely influences the composition of the resident bacterial communities in different environments (Maire et al., 2024; Turnham et al., 2021). Among colonies of the corals sampled, *P. acuta* and *M. foliosa*, bacterial taxa beta diversity varied significantly across habitats. Coral colonies in close proximity to human development had large differences in microbial community structure relative to conspecifics sampled from the nearshore habitat. However, *M. foliosa* was associated with *D. glynnii* at the nearshore habitat and *Cladocopium* C15 at the developed habitat, *while P. acuta* maintained the same association across these habitats; indicating coral species‐specific differences in Symbiodiniaceae and bacterial community associations across the nearshore and developed habitats. Because, *P. acuta* and *M. foliosa*, vertically transmit their Symbiodiniaceae symbionts (and some bacterial symbionts) to their eggs (Babcock et al., 1986; Kitchen et al., 2020; Penland et al., 2004), there is an increased chance of environmental mismatch for these coral species that may encourage dependence on their microbial communities for local acclimatisation (Botté et al., 2022). These two coral species also belong to families that are often more susceptible to disease (Acroporidae and Pocilloporidae) than others (Díaz & Madin, 2011; Palmer et al., 2010; Willis et al., 2004) and have many traits that increase their disease risk, such as complex growth forms, shallow depth range, large geographic range (Díaz & Madin, 2011), and low innate immunity (Palmer et al., 2010). Together, these factors combined with our results, highlight the importance of studying the acclimatisation potential of at‐risk coral species across a range of habitats (especially to anthropogenic stressors) to better understand stable and disturbed community dynamics for corals with high local microbial community acclimatisation.

### Conclusions

Across the three habitats explored in this study, colonies from the offshore and nearshore habitats exhibited few differences in microbial community structure, despite the nearshore habitat being warmer and more acidic than the offshore habitat and corals maintaining different Symbiodiniaceae associations across these habitats. These findings indicate that the coral holobiont can sustain stable symbiotic microbial relationships across varied environments. This resilience is likely achieved through the prolonged adaptation and acclimatisation of both the host and symbionts to local environmental conditions. An important exception to this pattern was observed in coral microbiomes near human development, where there were more pronounced disruptions in community structure than observed at the offshore and nearshore habitats. Numerous microbial taxa exhibited similar shifts in abundance across coral species from nearshore to developed habitats. These shifts may be linked to the fourfold increase in NH_4_^+^, which, while anecdotal, is commonly observed in urbanized areas, as well as higher NO_3_^−^ levels observed at the developed habitat. Other unmeasured anthropogenic factors associated with the developed habitat may also contribute to these changes. These findings suggest that for some coral communities, local anthropogenic factors may drive larger shifts in coral microbial associations than lower pH and increased seawater temperatures. Although Palau's inner bays are seen as potential refuges from climate change for Pacific corals (van Woesik et al., 2012), these observed changes in microbial communities and the impact of human proximity call for further investigation into how nearshore coral communities will be affected by continued climate change.

## AUTHOR CONTRIBUTIONS

**Shelby E. Gantt:** Conceptualization; investigation; writing – original draft; methodology; validation; visualization; writing – review and editing; formal analysis; data curation; software. **Keri M. Kemp:** Methodology; visualization; writing – review and editing; writing – original draft; formal analysis. **Patrick L. Colin:** Resources; writing – review and editing. **Kenneth D. Hoadley:** Resources; writing – review and editing. **Todd C. LaJeunesse:** Funding acquisition; methodology; investigation; writing – review and editing; resources. **Mark E. Warner:** Funding acquisition; investigation; methodology; writing – review and editing; resources. **Dustin W. Kemp:** Funding acquisition; investigation; conceptualization; writing – original draft; methodology; writing – review and editing; resources; supervision.

## CONFLICT OF INTEREST STATEMENT

The authors declare no conflict of interests.

## Supporting information


**Appendix S1:** Supplementary information.

**Appendix S2:** Supplementary information.

## Data Availability

ITS2 DNA sequences generated and analysedanalyzed during the current study are available in GenBank (accession numbers PP391597 – PP391614 and PP769353 – PP769369). 16S rRNA sequences used in this study are archived on the SRA database at accession number PRJNA1080224.
