# Supplementary material for: Influence of reef habitat on coral microbial associations
Source: Environ Microbiol Rep. 2024 Nov 8;16(6):e70051. doi: 10.1111/1758-2229.70051 (PMC11549029; doi:10.1111/1758-2229.70051)
Supplement: Supplementary file 2 — Appendix S2: Supplementary information. [file EMI4-16-e70051-s001.docx]

**Supplemental Figure 1.** Family-level relative abundance for individuals by coral species and habitat.

**Supplemental Figure 2.** PCoA of Bray-Curtis dissimilarities of seawater microbial communities by habitat. Habitats are indicated as follows: developed (yellow), nearshore (red), and offshore (blue). All ellipses are 95% confidence intervals and are colored by habitat. Pairwise P-values indicate significance of grouping by habitat with pairwise permutational analysis of variance (PERMANOVA). Ovals represent 95% confidence intervals of groupings. The developed habitat did not have enough replicates for ellipse creation, so no ellipse is included for these samples.

**Supplemental Figure 3.** Heat map of seawater microbial taxa that significantly differed in developed and offshore habitats compared to the nearshore habitat based on MaAsLin2 models. Taxa are broken up by taxonomy level analysis with results shown for within coral species across habitat comparisons. FDR controlled q-values greater than 0.05 are not included in the figure for clarity, while q-values lower than 0.001 are indicated with “< 0.001”. Red indicates taxa that are more abundant in the nearshore habitat (reference), white indicates no difference in abundance, and blue indicates taxa that are less abundant in the nearshore habitat.

**Supplemental Figure 4.** Heat map of *C. aspera* microbial taxa that significantly differed in developed and offshore habitats compared to the nearshore habitat based on MaAsLin2 models. Taxa are broken up by taxonomy level analysis with results shown for within coral species across habitat comparisons. FDR controlled q-values greater than 0.05 are not included in the figure for clarity, while q-values lower than 0.001 are indicated with “< 0.001”. Red indicates taxa that are more abundant in the nearshore habitat (reference), white indicates no difference in abundance, and blue indicates taxa that are less abundant in the nearshore habitat.

**Supplemental Figure 5.** PCoA of Bray-Curtis dissimilarities of coral microbial communities by dominant Symbiodiniaceae association. Dominant Symbiodiniaceae association are indicated as follows: *Cladocopium* spp. (blue), *Durusdinium* spp. (red).
